# Estimating Future Obstetrics and Gynecology Workforce Needs in Japan

**DOI:** 10.7759/cureus.78269

**Published:** 2025-01-30

**Authors:** Takashi Yoshimasu, Tasuku Inao, Isao Yokota

**Affiliations:** 1 Department of Biostatistics, Graduate School of Medicine, Hokkaido University, Sapporo, JPN

**Keywords:** 960-hour limit, concentration of hospitals, demographics, obstetrician and gynecologist, simulation study, work style reform

## Abstract

Introduction

The Ministry of Health, Labour, and Welfare of Japan (MHLW) introduced a regulation on physician overtime work in 2024, limiting overtime hours to a maximum of 960 annually. This regulation has raised concerns that the quality of medical care may decline. On the other hand, the shifting demographics of Japan, characterized by an aging population and declining birth rates, suggest a potential decrease in demand for obstetrics and gynecology (OBGYN) services in the future. In Japan, there is no feasible study regarding how many OBGYN doctors are currently lacking and how many of them will be necessary. Herein, we conduct simulations using Japanese insurance claims data to estimate the required number of OBGYN doctors.

Methods

This is a simulation study. We project future demand based on the current demand, working hours, and demographic changes. Using health insurance claims data, we estimate how many OBGYN doctors will be necessary in the future within the overtime work limit.

Results

The required number of doctors shows a declining trend that mirrors the declining population. Presently, there is a shortage of 1,500 doctors to comply with the 960-hour limit, and it is anticipated to be until 2040 or later before all doctors adhere to this limit. Several sensitivity analyses consistently support this trend.

Conclusions

Despite the existing shortage of obstetricians and gynecologists, future demands are expected to decrease. Inter-hospital and regional communication should be promoted for systemic innovations.

## Introduction

The Ministry of Health, Labour, and Welfare of Japan (MHLW) implemented the regulation of overtime work in 2024, limiting overtime hours to a maximum of 960 per year [1]. However, concerns have been raised, mainly by senior physicians, that the regulation could negatively impact the quality of care in Obstetrics and Gynecology (OBGYN) due to reduced training opportunities [2]. To address the current medical demand under these regulations, temporary measures such as "Night and Holiday Duty Permission" have been introduced to make physicians' working hours appear shorter than they actually are [3].

Meanwhile, the demographics of Japan are changing due to an aging population and declining birth rates. As the population ages, the demand for healthcare services for elderly women, including treatments for malignancies, is expected to rise. Conversely, declining birth rates and population numbers are anticipated to reduce the demand for perinatal medicine.

Amid these significant changes, it is crucial to estimate how many OBGYN doctors are currently lacking and how many will be needed in the future to develop effective health policies for Japanese OBGYN doctors. In response to this need, the Japan Society of Obstetrics and Gynecology (JSOG) proposed the “Grand Design,” recommending the recruitment of 500 OBGYN residents annually [3]. However, this target lacks epidemiological evidence to support it. While acknowledging the declining birth rates, the society predicts an increase in high-risk pregnancies due to the aging of pregnant women, the growing focus on women’s healthcare, and medical innovations such as robotic surgery, genomic medicine, and personalized healthcare. Therefore, the society argues it is premature to reduce the number of OBGYN doctors [3]. This underscores the urgent need for robust predictions regarding the future demand for OBGYN doctors in Japan.

Predicting the required number of doctors is inherently challenging and has been a topic of study since 1978 [4]. Estimation methods generally focus on supply, demand, or a combination of both [5]. Supply-based estimation considers factors such as the inflow and outflow of physicians, including medical school graduates, immigration, retirements, attrition, and physicians working abroad [5,6]. In contrast, demand-based estimation involves factors such as population size (demography), morbidity, healthcare utilization patterns, and growth in health expenditures [5]. However, few studies have specifically focused on predicting the future demand for OBGYN doctors [7,8]. These reports suggest that future demand would increase in response to the population growth of women of reproductive age (18-44 years old), based on a baseline scenario that assumes healthcare demands remain constant as long as demographic trends remain unchanged [7,8].

In this study, we estimate the future required number of OBGYN doctors using a demand-based approach and a baseline scenario, focusing on the radically changing demographics of Japan and the new regulations of overtime work.

## Materials and methods

This study is a simulation using insurance claims data and demographic information. The simulation consists of four steps. The first step is to analyze the current demand by quantifying the workload of OBGYN doctors. Second, we evaluate the current number of OBGYN doctors and their working hours. Third, we estimate the future demand based on projected demographic changes. Finally, we calculate the number of OBGYN doctors required to meet the demand. All analyses were performed using R (version 4.2.3, R Foundation for Statistical Computing, Vienna, Austria).

Current demand

**Figure 1:**
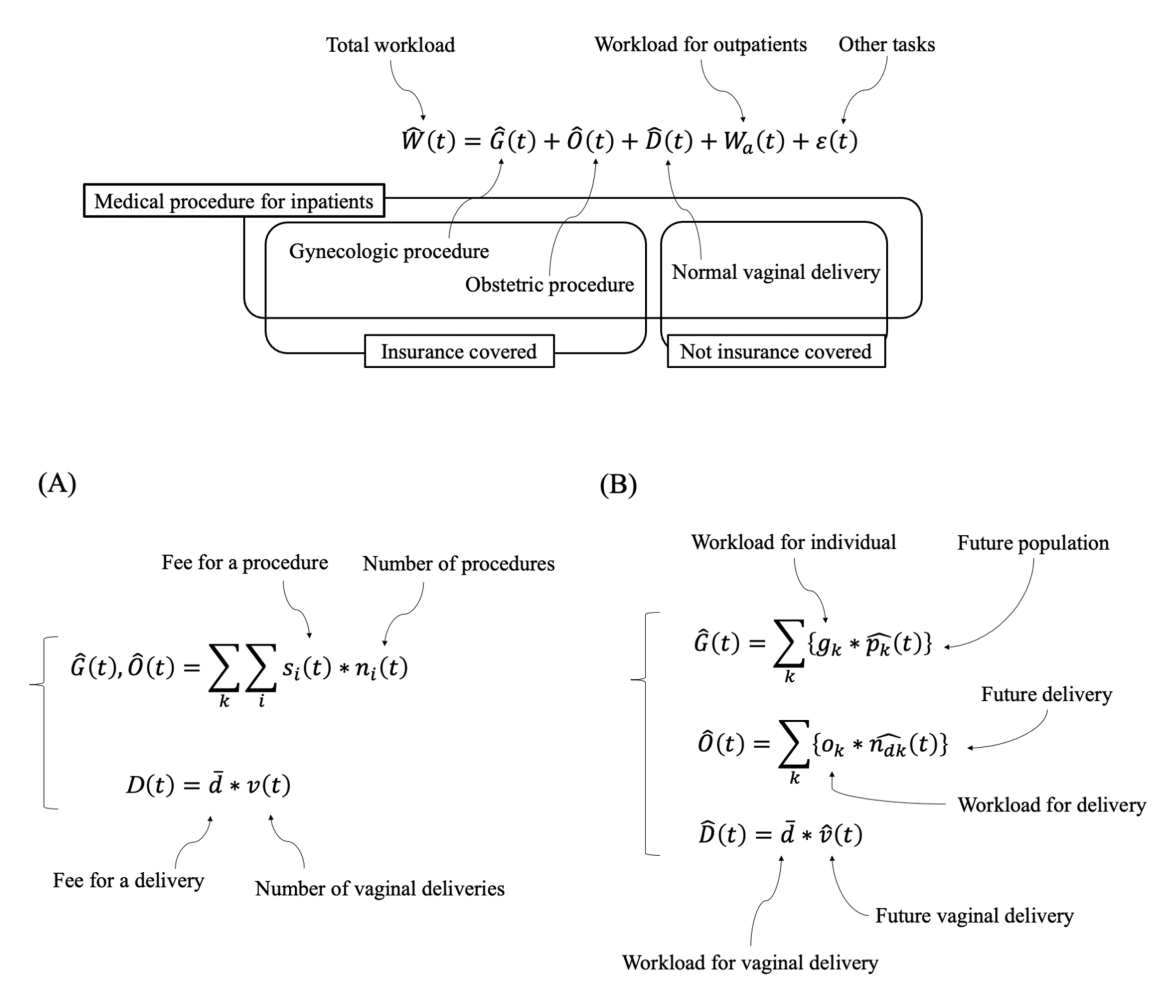
Total workload The total workload comprises three components: medical procedures for inpatients, workload for outpatients, and other tasks. We focus on medical procedures for inpatients, which include both procedures covered by insurance and those not covered by insurance. (A) describes how we quantify the current demand by using medical procedures for inpatients. (B) describes how we estimate future demand based on demographic changes. Note that $\widehat{n_{dk}}(t)$ represents the number of deliveries in year $t$, including cesarean deliveries, whereas $\widehat{v}(t)$ represents the number of deliveries in year $t$, excluding cesarean deliveries.

The total workload of OBGYN doctors consists of three main components: medical procedures for inpatients, workload for outpatients, and other tasks (Figure 1). Unfortunately, no comprehensive data exists to capture the total workload. Estimating the workload for outpatients is particularly challenging due to the diverse range of activities involved, such as ultrasound examinations, prescribing medications, and providing informed consent regarding patients' conditions. These activities are difficult to quantify with health claims data. Other tasks include attending conferences, writing electronic medical records and letters, and conducting research, all of which are difficult to quantify.

As a result, only the workload associated with medical procedures for inpatients can be quantified. These procedures include gynecologic procedures, obstetric procedures, and those related to normal vaginal deliveries. Gynecologic procedures include laparoscopic surgery and laparotomy, while obstetric procedures consist of colpo-perineal sutures, vacuum extractions, and cesarean sections. Procedures for normal vaginal deliveries involve cardiotocogram monitoring, internal examinations, and waiting hours. Among these, gynecologic and obstetric procedures are covered by insurance, whereas normal vaginal deliveries are not. For the purpose of this study, we assume the workload for each procedure is proportional to its medical fee. For normal vaginal deliveries, which are not covered by insurance, we use an alternative fee as described below.

Supply

In this study, we assume that the age structure and geographic distribution of OBGYN doctors will remain constant over time. Additionally, we define $h(t)$ as the total working hours in year $t$. The following equation is used to represent efficiency:

$r=\frac{G(t)+O(t)+D(t)}{h(t)}$

In this equation, $r$ represents the workload of procedures for inpatients per unit time, indicating efficiency. $G(t)$, $O(t)$, and $D(t)$ represent gynecologic procedures, obstetrics procedures, and procedures for normal vaginal deliveries, respectively.

Future demand

To project future demand, we use the workload per individual and per delivery (Figure 1). The workload is estimated based on data collected from 2015 to 2021. While the number of gynecologic procedures is assumed to change proportionally with the population size, the number of obstetric procedures varies according to the number of deliveries, including cesarean sections. Similarly, the number of procedures for normal vaginal deliveries is determined by the number of vaginal deliveries, excluding cesarean sections. Additionally, we assume that the workload associated with outpatients and other tasks changes in proportion to the volume of medical procedures for inpatients.

Future OBGYN doctors

In Japan, statutory working hours are 8 hours per day and 40 hours per week [9]. With 16 national holidays annually and mandatory paid leave of five days [10], the statutory working hours for each physician amount to 1,917 hours per year.

The MHLW has proposed regulations on overtime work, setting an upper limit of 960 hours per year. An extended limit of 1,860 hours applies exclusively to physicians working in underserved healthcare areas, emergency medicine, and those in training. However, this 1,860-hour limit is set to expire in 2035 [1]. Furthermore, information regarding part-time work is not available for those affiliated with gynecologic hospitals or clinics without inpatient beds [11]. Since they work fewer hours than the statutory working hours at their affiliated facilities [11], we assume they are not engaged in overtime work. However, this assumption is further evaluated in a sensitivity analysis. Importantly, the regulation does not apply to OBGYN doctors who manage labor-handling clinics, as they are considered employers rather than employees. In this regard, we assume that all doctors, except for those affiliated with labor-handling clinics, will work an additional 960 hours of overtime annually in the future.

Sensitivity analysis

Firstly, since the working hours of OBGYN doctors vary significantly, the regulation will reduce the average overtime work to 960 hours or fewer annually. Therefore, we assume three additional scenarios for the average overtime work of future OBGYN doctors who currently exceed the 960-hour limit: (A) no overtime work, (B) 360 hours annually, and (C) 720 hours annually.

Secondly, as mentioned above, we assume that the hours dedicated to part-time work are zero for those affiliated with gynecologic hospitals or clinics without inpatient beds. We consider an additional four scenarios for part-time work of this population: (A) 0 hours annually, (B) 360 hours annually, (C) 720 hours annually, (D) 960 hours annually, and (E) 1860 hours annually. In these scenarios, we assume that the hours of overtime work remain unchanged for scenarios (A), (B), (C), and (D). In scenario (E), however, we assume overtime work of 960 hours annually starting after 2025. Scenario (A) is used for the primary analysis in this study.

Finally, we apply different $\overline{d}$ values, representing the workload for a single vaginal delivery.

Data source

Current Demand

We utilize the National Database of Health Insurance Claims and Specific Health Checkups of Japan (NDB open data Japan) from the year 2015 to 2021 [12]. From this dataset, we extract medical procedures from the inpatient data categories “J medical procedures” and “K surgeries,” which are associated with OBGYN doctors. Codes of the Japanese health insurance system used in this study are summarized in Appendix A.

Although infertility treatments became eligible for insurance coverage in April 2022 [13], specific data on these treatments are not currently available. These treatments, which are primarily conducted on an outpatient basis, are not included in this analysis, though they may affect the future demand for OBGYN services.

Additionally, based on a previous study on childbirth expenses, we estimate the average delivery fee to be 257,981 Japanese yen, equivalent to 25,798.1 points when covered by insurance [14]. This value is used for $\overline{d}$. The annual number of deliveries was sourced from demographic surveys [15].

Supply

In this study, we use in-hospital time as a measure of working hours. Data on the number of OBGYN doctors in each hospital and their working hours are sourced from the annual survey by the Japan Association of Obstetricians and Gynecologists (JAOG) [11]. This survey covers all 5,230 facilities with OBGYN departments, with responses from 5,146 facilities, resulting in a response rate of 98.4%.

Additionally, part-time work is calculated using data from a JAOG questionnaire survey in 2021 [16]. This survey targeted 985 birthing hospitals, excluding clinics, and received responses from 715 facilities, achieving a response rate of 72.6%. The annual total in-hospital hours are determined by combining daytime duty hours (8 hours per duty) and overnight duty hours (16 hours per duty) from other hospitals. Unfortunately, there is no available data on part-time work for OBGYN doctors employed in gynecologic hospitals and clinics. As a result, this study does not account for part-time work by these doctors. The average values for 2020 and 2021 are used as the baseline for efficiency, as the total in-hospital hours are calculated using data from mid-2021.

Future Demand

To estimate future demand, we use population data from the Statistics Bureau of Japan [17] and population projections from the National Institute of Population and Social Security Research [18-20]. These projections are based on the national population census conducted in 2020 and were published in 2023.

Assumptions

Assumptions used in this study are summarized in Appendix B.

## Results

Current in-hospital hours

The total in-hospital hours are shown in Table 1. Note the regulation will reduce the working hours of doctors in hospitals, but not in labor-handling clinics. Since data regarding part-time work is not fully available, the total in-hospital hours may underestimate the actual hours. The total workload, denoted as $W(t)$, is distributed among 11,976 OBGYN doctors as shown in the table. Given a response rate of 98.4%, the actual number is estimated to be 12,171 doctors.

**Table 1:** Total in-hospital hours across different facilities ^†^: these values have no data, and are treated as zero; ^‡^: the working hours in clinics without beds are those of clinics that do not handle infertility treatments; ^§^: these values are not defined

Facility type	Number of doctors	Number of facilities	Overtime work (hours)	Work in other facilities (hours)	Total in-hospital hours	Total hours
Comprehensive perinatal center	1,819	112	1,141	874	3,932	7,151,580
Local perinatal center	2,309	294	1,157	864	3,938	9,092,842
General hospital (handling labor)	2,471	587	924	970	3,811	9,415,993
Labor-handling clinic	1,919	1,173	2,847	^†^	4,764	9,142,116
Gynecologic hospital	627	447	-111	^†^	1,806	1,132,362
Clinic without beds	2,831	2,364	-441^‡^	^†^	1,476	4,178,556
Total	11,976	4,977	^§^	^§^	^§^	40,113,449

Current and future demand

**Figure 2:**
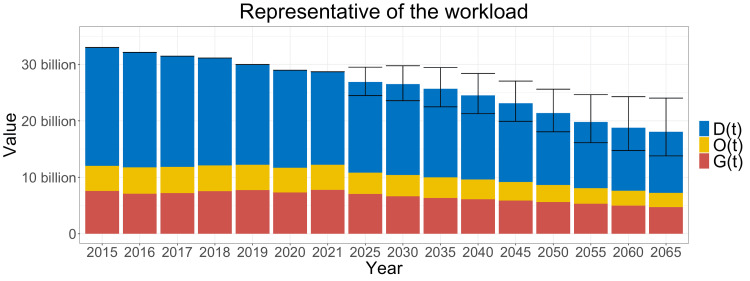
Current and future demands This graph illustrates the current demands and future projections, scaled using insurance reimbursement points. $G(t)$, $O(t)$, and $D(t)$ are the total workload of gynecologic procedures, the total workload of obstetric procedures, and the workload of vaginal deliveries not covered by insurance, respectively. These three components serve as representatives of the total workload. Note that the values before 2021 reflect the current data, while those after 2025 are future estimations. The maximum value of this graph corresponds to a population projection based on high birth rates and low death rates, while the minimum value of this graph corresponds to a population projection based on low birth rates and high death rates. The workload demonstrates a decreasing trend over time.

Medical procedures for inpatients are shown in Figure 2. The current demand and the future estimation are on the decline, mainly due to the decrease in childbirth. As this projection is based on the national population census in 2020, the birth rates have a wide range of estimation since 2025.

Future OBGYN doctors

**Figure 3:**
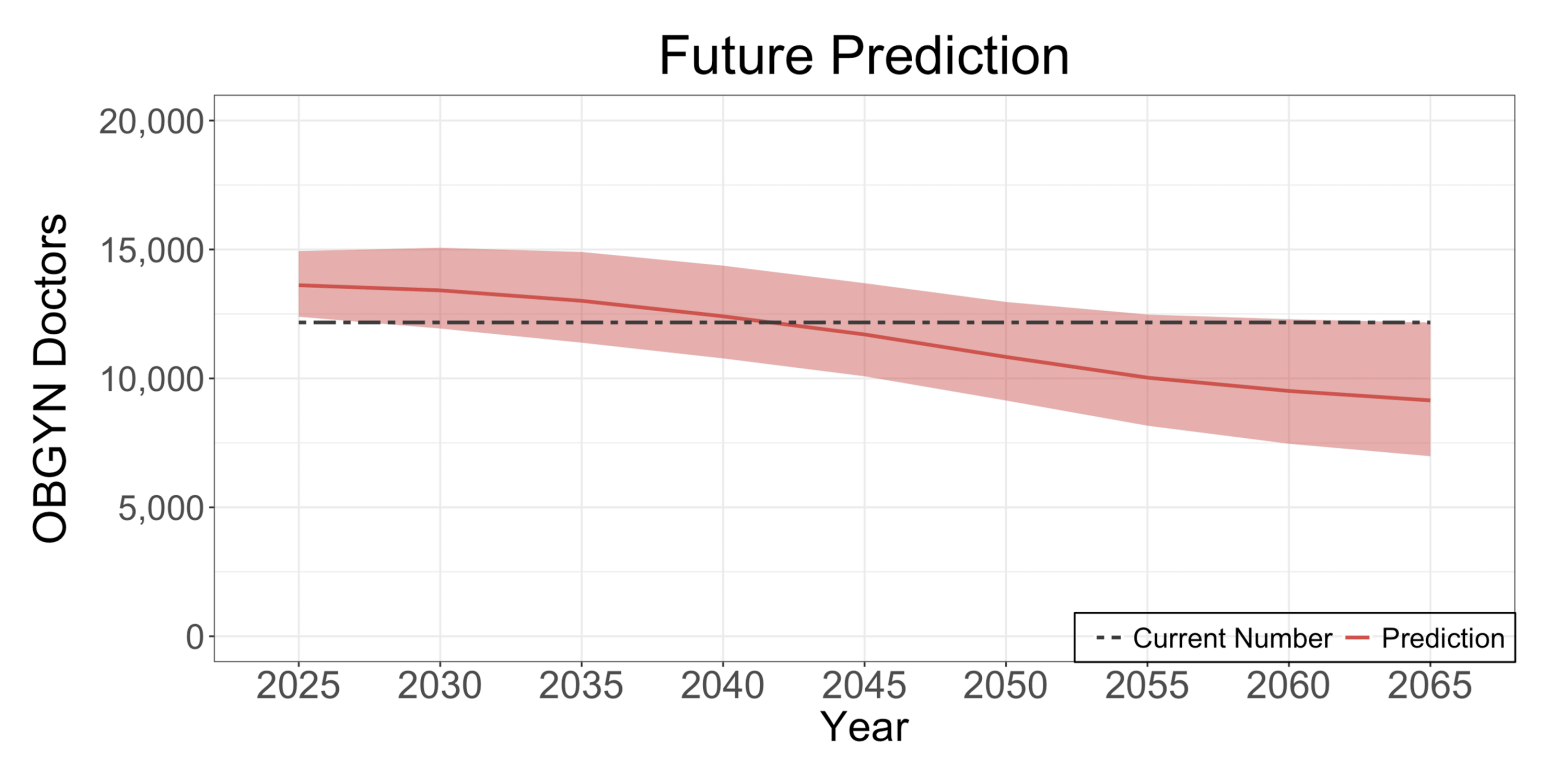
The future required number of OBGYN doctors The curve represents the predicted number of OBGYN doctors needed in the future. The range of the curve corresponds to demand, with the upper limit reflecting the scenario of the lowest mortality rate and highest birth rate and the lower limit corresponding to the scenario of the highest mortality rate and lowest birth rate. The black dashed line shows 12,171, which is the current number of doctors maintaining the workload. OBGYN: obstetrics and gynecology

The required number of OBGYN doctors is shown in Figure 3. This number declines over time, reflecting decreasing birth rates and a shrinking younger generation. The estimation corresponds to a workload currently managed by 12,171 doctors. Therefore, approximately 1,500 additional OBGYN doctors are needed to achieve the 960-hour limit. Without intervention, it will take until 2040 or later for all OBGYN doctors to achieve this limit.

Sensitivity analysis

**Figure 4:**
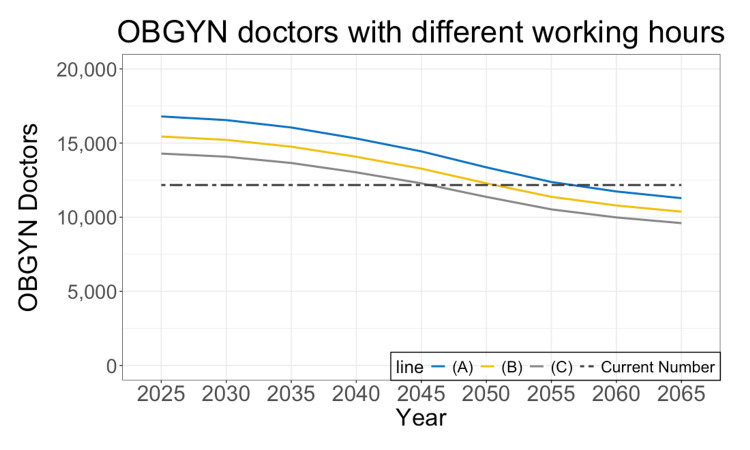
Future prediction with different working hours The figure shows the required number of OBGYN doctors. There are three scenarios in this figure: (A) no overtime work, (B) 360 hours of overtime work, and (C) 720 hours of overtime work. The black dashed line shows 12,171, which is the current number of doctors maintaining this workload. OBGYN: obstetrics and gynecology

**Figure 5:**
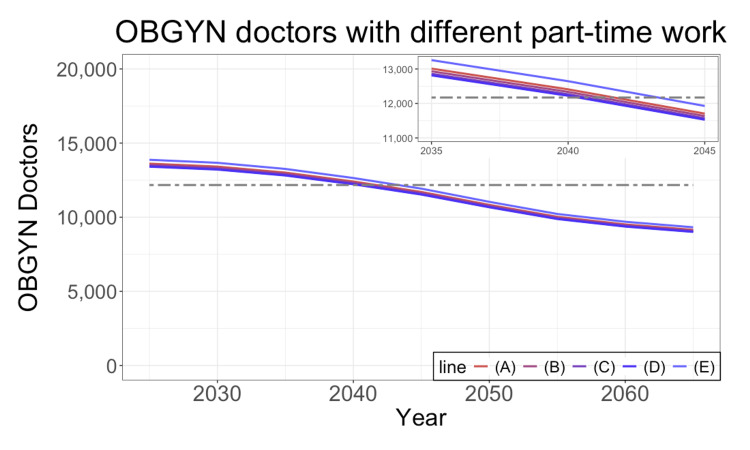
Future prediction with different part-time work The figure shows the required number of OBGYN doctors. We assume five scenarios for the average hours of part-time work for physicians affiliated with gynecologic hospitals or clinics without inpatient beds: (A) 0 hours, (B) 360 hours, (C) 720 hours, (D) 960 hours, and (E) 1860 hours. (A) corresponds to the estimates in Figure 3. The black dashed line shows 12,171, the current number of OBGYN doctors. The inset in the upper right corner enlarges a portion of the main figure. OBGYN: obstetrics and gynecology

**Figure 6:**
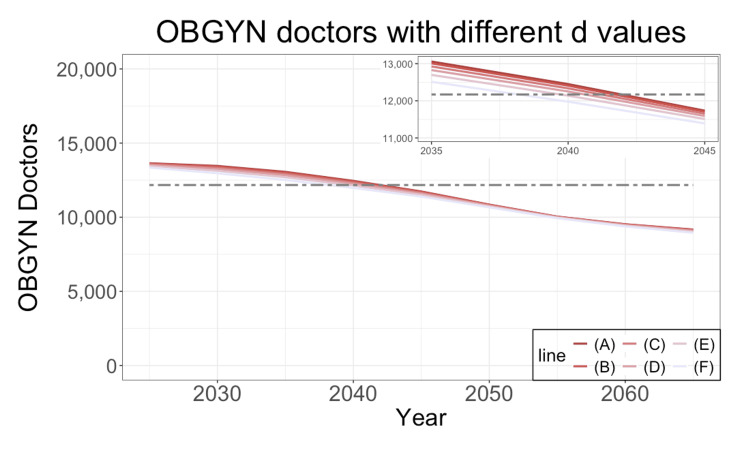
Future prediction with different values for each delivery This figure shows the required number of OBGYN doctors with different $\overline{d}$ values, the workload for each delivery. This number corresponds to the scenario where OBGYN doctors work up to 960 hours overtime. There are six assumptions in this figure: (A) 30,000 points, (B) 25,171 points, (C) 20,000 points, (D) 15,000 points, (E) 10,000 points, and (F) 5,000 points. (B) corresponds to the estimates in Figure 3. The black dashed line shows 12,171, the current number of OBGYN doctors. The inset in the upper right corner enlarges a portion of the main figure. OBGYN: obstetrics and gynecology

Several sensitivity analyses are presented in Figures 4-6. Figure 4 shows various scenarios based on different average overtime hours. These predictions use a population projection based on medium birth rates and medium death rates. If the average overtime work is 720 hours annually, there is currently a shortage of 2,000 doctors; however, the current number will be sufficient until 2045. By 2055 to 2060, all OBGYN doctors are expected to achieve no overtime work.

Figure 5 presents various scenarios based on different assumptions regarding part-time work for physicians affiliated with gynecologic hospitals or clinics without inpatient beds. These predictions are based on population projections using medium birth rates and medium death rates. If the average hours of part-time work for this population remain within 960 hours, the assumption used in this study, as represented in Figure 3, does not significantly affect the estimation. However, if the average hours for part-time work are assumed to be 1860 hours, the estimation indicates a slightly higher required number of OBGYN doctors.

Figure 6 shows the required number of OBGYN doctors assuming an overtime workload of 960 hours under different assumptions. If the workload for a single vaginal delivery, denoted as $\overline{d}$, is actually lower than 25798.1 points, the 960-hour limit could be achieved a few years earlier than estimated.

## Discussion

This study aims to estimate the future required number of OBGYN doctors using Japanese insurance claims data. This estimation focuses on current demand and demographic changes to project future demands. For simplicity, we based the estimation on the changes in the workload for inpatients. To represent the total workload of OBGYN doctors, we considered medical procedures covered by insurance as well as normal deliveries not covered by insurance. Although OBGYN doctors are currently in short supply, the required number of OBGYN doctors is expected to decline in the future due to decreasing population and birth rates. While this study relies on several assumptions, our sensitivity analyses consistently demonstrate this trend.

In Japan, many small labor facilities heavily rely on part-time OBGYN doctors who have primary affiliations elsewhere [11]. JAOG has expressed concerns that the regulation of overtime work will discourage part-time work in smaller labor facilities, potentially destabilizing perinatal medical services. Consequently, JAOG has recommended that labor facilities obtain “overnight and holiday duty permission” [3]. When overnight and holiday duty in certain facilities requires minimal tasks for physicians, hours worked are not counted as working hours unless actively performing medical procedures [21]. With this permission, labor facilities can invite OBGYN doctors for part-time work without caring about the 960-hour limit. However, it may lead to prolonged and unfair working hours. Therefore, JAOG considers this permission as only a temporary measure [3]. Therefore, we use in-hospital time as working hours, which includes sleep and waiting time, because in-hospital time will be fully counted as working hours in the coming years.

The regulation will reduce working hours. However, working hours may vary significantly within the same facility, and the average overtime work after the regulation may not strictly align with the 960-hour limit. Thus, we anticipated an additional three scenarios, as shown in Figure 4. If the average overtime work becomes 720 hours, all OBGYN doctors achieve the 960-hour limit in 2045 or later. Additionally, OBGYN doctors in labor-handling clinics work for incredibly long hours (Table 1). Though the regulation does not intend to limit overtime work for those who manage labor-handling clinics, the younger generations are not likely to inherit these long working hours, judging from their attitude toward work [2]. Therefore, the estimates based on these hardworking OBGYN doctors (Figure 3) may undervalue the future required number of OBGYN doctors.

As detailed information on part-time work is not fully available, we conducted a sensitivity analysis for part-time work. In Figure 5, the average hours dedicated to part-time work have minimal impact on the estimates as long as they remain within 960 hours.

Moreover, we established a standard fee for normal vaginal deliveries, which requires sensitivity analysis. In Figure 6, a smaller workload leads to achieving the 960-hour limit earlier. If $\overline{d}$ is 5,000 points under insurance coverage, the 960-hour limit will be achieved in 2038 or later.

The main assumption in this estimation is that healthcare supply will remain unchanged if demographics remain the same. However, this baseline scenario may not always hold true due to medical innovations, an increase in female physicians, changes in the allocation of physicians, and advanced data sharing.

In this study, we did not take gender differences into account. In Japan, female doctors tend to work fewer hours than male doctors because they are engaged in parenting [22]. Female doctors occupy approximately 40% of OBGYN doctors [23], and this proportion is increasing [22]. JSOG and JAOG have been calling for action to ease burdens on female doctors [3,16]. However, we will need more OBGYN doctors than our estimates if the surrounding environment of female doctors does not improve sufficiently and female doctors continue to increase.

The estimates are all based on the current medical system. Japan has an excessive number of labor facilities, approximately 2,000 nationwide [11]. The average delivery per hospital is 475 deliveries annually, with 7.4 OBGYN doctors per hospital on average [16]. Many labor facilities have fewer than two physicians, and most clinics are assumed to be managed by only a small number of doctors [16]. Japan's medical and human resources are inefficiently dispersed compared to countries like the United States and Finland, where hospitals are highly concentrated [24,25]. This decentralized system results in an excessive number of OBGYN doctors being on standby during nights and holidays.

Maternal transfers from smaller clinics to larger hospitals are common due to the limited capacity of many clinics. These transfers pose significant risks to patients, as a recent study in Japan found that the average transfer takes approximately one hour to reach a tertiary center [26]. This highlights the inherent inefficiencies and risks of the current dispersed system. Moreover, physicians are often unable to rest properly at night, as they remain prepared for potential emergencies. Addressing these systemic issues is crucial to improving both efficiency and patient safety.

In addition to these structural inefficiencies, there are also issues with personal data sharing. While the exchange of patient records between hospitals has progressed in countries such as the United States and Singapore, inter-hospital communication in Japan still predominantly relies on telephone, fax, and paper-based medical records [27]. This system is not only inefficient but also poses risks, as it increases the likelihood of overlooking critical information. By promoting personal data sharing, physicians could dedicate more time to patients, potentially reducing overall demands.

Therefore, we should prioritize deliveries in well-equipped hospitals initially and promote personal data sharing. In centralized facilities, more deliveries can be managed per in-hospital hour, reducing waiting times. Additionally, large hospitals are reported to have better neonatal outcomes [28-30]. In terms of neonatal outcomes as well, consolidating birth facilities should be encouraged. Small clinics should focus more on outpatient care rather than taking risks with deliveries. The redistribution of human resources from small clinics to larger hospitals will also be necessary. By concentrating deliveries and surgeries in larger hospitals, the issue of low physician salaries in teaching hospitals could be addressed [31]. In short, if we were to maintain the current number of birth facilities, more OBGYN doctors would be required than our estimates suggest, as efficiency would decline with decreasing birth rates.

However, neonatal outcomes are significantly worsened in cases of out-of-hospital births. If travel time to the hospital takes long, out-of-hospital births become a serious concern [24,32], indicating that hospital concentration should be carefully planned.

Ultimately, we should focus on meaningful dialogue with stakeholders to create a better medical system for both patients and physicians. This is far more constructive than devising ways to manipulate working hours to circumvent regulations for "overnight and holiday duty permissions."

Strengths and limitations

The methods used in this study quantify the workload based on medical fees and use demographic changes to make estimations. These methods can be applied as an alternative in other fields, particularly when crucial information is unavailable. Several sensitivity analyses also support the robustness of the results in this study. However, the assumptions made in this study may introduce bias into the estimates. The primary limitation of this study is that we could not quantify outpatient workload to predict the future demand for OBGYN doctors. This may introduce bias; if treatment trends shift toward more conservative management in outpatient settings, the actual demand for physicians may be lower than our estimates. Conversely, with the increasing demand for infertility treatment, more patients may seek medical assistance through outpatient clinics. In this case, our study would likely underestimate the required number of physicians. Additionally, changes in the efficiency of medical services should be taken into account. If the current number of birth facilities is maintained, more OBGYN doctors will be required. Furthermore, increasing demands for innovative medical treatments could distort the projections, though the extent of these changes remains uncertain.

## Conclusions

Using claims data, we estimated the required number of OBGYN doctors based on medical procedures and vaginal deliveries, highlighting declining trends. Our analysis indicates a shortage of 1,500 doctors for a 960-hour overtime work limit. However, the current workforce is expected to be sufficient until around 2040. Simply increasing the number of OBGYN doctors will not be enough to address the current excessive workload and the projected decline in demand. Instead, we should prioritize inter-hospital and regional communication and collaboration, rather than merely relying on the long working hours of physicians. Structural reforms must be explored more extensively, including the concentration of hospitals and clinics, improvement of working conditions for healthcare workers, and the digitalization of medical information.

## Appendices

Appendix A

The procedure codes of the Japanese health insurance system are provided for replicability.

**Table 2:** Procedure code The codes of the Japanese health insurance system are listed here. The number of gynecologic procedures is adjusted in proportion to the population size, while the number of obstetrics procedures is adjusted in proportion to the number of deliveries.

	Codes
Gynecologic procedure code of Japanese health insurance system	140028010, 140015110, 140015210, 140015410, 140015510, 140015710, 140015910, 140016010, 140016150, 140016210, 140016310, 140016510, 140017110, 140056310, 140056410, 140017210, 150209510, 150209610, 150210510, 150210610, 150210710, 150210910, 150211010, 150211110, 150211350, 150211410, 150211510, 150326610, 150211650, 150211750, 150211810, 150211910, 150212010, 150212110, 150326710, 150212310, 150326810, 150212410, 150212810, 150212910, 150213010, 150213150, 150190550, 150190650, 150213210, 150404510, 150404610, 150213610, 150213710, 150213810, 150213950, 150278410, 150285910, 150286050, 150365810, 150214510, 150326910, 150214610, 150264510, 150293910, 150294010, 150365910, 150214910, 150215010, 150215110, 150215210, 150215310, 150215410, 150390410, 150421210, 150216010, 150216150, 150216510, 150002150, 150281950, 150216650, 150327010, 150216810, 150216910, 150278510, 150294110, 150421310, 150421410, 150421510, 150421610, 150281750, 150421710, 150421810, 150217410, 150366010, 150217510, 150272250, 150409110, 150409210, 150217610, 150327210, 150217710, 150379810, 150409310, 150409810, 150218210, 150218310, 150218410, 150218710, 150218850, 150218950, 150219010, 150219210, 150379910, 150219410, 150264610, 150219710, 150264710, 150219850, 150267650, 150219650, 150267750, 150299850, 150220010, 150270010, 150409510, 150421910, 150422010, 150220150, 150220250, 150220450, 150268050, 150268150, 150268250, 150220710, 150220910, 150282050, 150366110, 150224210, 150404710, 150404810, 150306750, 150224410, 150224510, 150264910, 150349310, 150349410
Obstetrics procedure code of Japanese health insurance system	140015610, 140015810, 140016410, 140016610, 140017010, 140017310, 140017450, 140017510, 140039110, 150221010, 150221110, 150221210, 150221310, 150221410, 150221510, 150221610, 150221710, 150221810, 150221910, 150222010, 150222110, 150222210, 150222310, 150222410, 150222550, 150222610, 150222710, 150222810, 150222910, 150223010, 150223110, 150223210, 150223310, 150242350, 150327310, 150223410, 150223510, 150223610, 150223710, 150223910, 150224010, 150366210, 150368250, 150410350, 150422110, 150390570

Appendix B

A summary of the assumptions used in this study is provided below. 

**Table 3:** Summary of assumptions

Assumptions
The workload for each medical procedure is equal to its medical fee.
The workload for one delivery, represented as d, is 25,798.1 points.
The proportion of the workload to the total in-hospital hours of OBGYN doctors is constant.
The age structure and facility distribution of OBGYN doctors will remain constant.
Gynecological procedures are proportional to the population.
Obstetrical procedures are proportional to delivery counts.
The workload for each individual and delivery will remain constant.
Cesarean delivery rates are the average cesarean rates from 2015 to 2021.
OBGYN doctors in labor-handling clinics and gynecologic hospitals do not engage in part-time work outside.
